# Hot-Melt Extrusion Enhances Antioxidant Effects of Mulberry on Probiotics and Pathogenic Microorganisms

**DOI:** 10.3390/antiox11112301

**Published:** 2022-11-21

**Authors:** Eun Ji Go, Byeong Ryeol Ryu, Gyeong Ju Gim, Ha Yeon Lee, Han Sol You, Hyun Bok Kim, Hyun Tai Lee, Ji Young Lee, Man Sop Shim, Jong-Suep Baek, Jung Dae Lim

**Affiliations:** 1 Department of Bio-Health Convergence, Kangwon National University, Chuncheon 24341, Korea; 2 Department of Herbal Medicine Resource, Kangwon National University, Samcheok 25949, Korea; 3 National Institute of Agricultural Sciences, RDA, Wanju 55365, Korea; 4 Division of Applied Bioengineering, Dongeui University, Busan 47940, Korea; 5 Department of Opthalmology and Visual Science, The Catholic University, Seoul 06591, Korea; 6 BeNatureBioLab, Chuncheon 24206, Korea

**Keywords:** mulberry, anthocyanin, hot-melt-extrusion, water solubility, release, probiotics, intestinal microbial flora, bioavailability

## Abstract

Mulberry is a rich source of anthocyanins (ACNs) known to possess biological activities. However, these ACNs are unstable in high pH, heat, and aqueous environments with a low bioavailability. In this study, a colloidal dispersion was prepared by hot melt extrusion with proper excipients. In this process, a hydrophilic polymer matrix was used to confirm the stability of the compound in high pH, high temperature, and aqueous media. It was confirmed that the particle size and the polydispersity index value were reduced, thereby improving the solubility. In vitro release studies revealed that the extrudate had a sustained release compared to a non-extruded product. As a result of measuring changes of intestinal microorganisms (*Lacticaseibacillus rhamnosus*, *Pediococcus pentosaceus*, *Escherichia coli, Enterococcus faecalis*, and *Staphylococcus aureus*), contents of probiotics were found to be increased whereas contents of pathogenic microorganisms were decreased. Thus, hot-melt extrusion could enhance the stability of ACN with prolonged release. The processed formulation exhibited probiotic properties and antimicrobial activities against pathogenic intestinal microflora.

## 1. Introduction

Anthocyanins (ACNs) are water-soluble flavonoids present in the phenolic system and have functional group bonds between sugar moieties [1]. Recently, there have been many reports on the improvement/development of industrial colorants, health-functional foods, and supplements [2]. Mulberry contains cyanidin-3-glucoside and cyanidin-3-rutinoside as major ACNs [3]. The content of ACNs in mulberry is 20 times higher than in grapes [4]. ACNs as natural pigments are mainly present in fruits [5]. Various studies have shown that ACNs have anti-inflammatory, anti-cancer and homeostatic effects [6,7]. 

Health-promoting effects of ACNs have been shown to be related to regulation of gut microbiota [8]. ACN metabolism can control the growth of certain bacteria due to their microbial metabolites [8]. In this sense, ACNs lower intestinal pH and increase short-chain fatty acid (SCFA)-producing bacteria that can inhibit the growth of pathogenic bacteria by regulating the gut microbiome. SCFAs improve the intestinal barrier, preventing the migration of pathogens and antigens [9]. Thus, we can regulate intestinal microflora by increasing the bacterial fermentation process and, thereby, increasing metabolite production [10].

Healthy intestines have effective digestion and absorption functions in the gastrointestinal tract and do not have chronic diseases, such as gastrointestinal diseases and colorectal cancer. Balanced gut microbiota has also been implicated in effective immune system function required to maintain host homeostasis [11]. Gut microbiota consists of more than one trillion microorganisms that have established symbiotic relationships with the host. These microbes can help keep the intestinal barrier intact by preventing colonization of potentially pathogenic microbes and modulating the mucosal immune system [12]. Thus, the barrier of epithelial cells and mucous layers provides a line of defense by various mechanisms, such as microbes, antibodies, and mucus production [11]. Damage to this barrier leads to inflammatory bowel disease, infection, and impaired immune metabolism, leading to disease [13]. Plants and bioactive substances of plant origin have beneficial effects on the intestinal wall, thereby reducing the risk of diseases by showing beneficial effects on intestinal health.

Although there are many known techniques for coating with various antimicrobial drug delivery systems [14,15,16], there is a need for an antimicrobial polymer technology that integrates HME (Hot Melt Extrusion) process and antimicrobial materials into polymers. To solve this problem, a functional material having low water solubility was prepared as an amorphous solid dispersion (SD) [17]. The SD is a promising strategy for increasing water solubility by dispersing hydrophobic molecules in carriers, such as polymers and sugar alcohols [18].

The use of low-water soluble amorphous food ingredients can dramatically improve gastric release [19]. HME provides sustained release by preparing a matrix of antibacterial agents for various cosmetics and pharmaceuticals purposes [20]. The long-term release of antimicrobial agents is scalable. It can produce custom antimicrobial polymer systems (HME of photodynamic antimicrobial polymers for the prevention of microbial contamination). Therefore, this study seeks to emphasize the potential of HME through the long-term release of mulberry ACNs, which have antibacterial properties, through the HME process.

## 2. Materials and Methods

### 2.1. Materials

The caffeic acid, keracyanin chloride, kuromanin chloride, Folin-Ciocalteau, and quercetin used in the experiment were purchased from Sigma-Aldrich (St. Louis, MO, USA). Aluminum chloride hexahydrate and potassium acetate were purchased from Junsei Chemical (Tokyo, Japan). Citric acid, sodium alginate, mannitol, and poloxamer 188 were purchased from Daejung (Seoul, Republic of Korea). Whey protein isolate, lecithin, and ascorbyl palmitate were purchased from ESfood (Gumpo, Republic of Korea). *Lacticaseibacillus rhamnosus* (*L. rhamnosis*), *Pediococcus pentosaceus* (P. pentosaceus), *Enterococcus faecalis* (*E. faecalis*). *Escherichia coli* (*E. coli*), and *Streptococcus aureus* (*E. aureus*) were purchased from ATCC (American Type Culture Collection). Man-Rogasa-shape (MRS) and brain heart infusion (BHI) medium was purchased from Solarbio (Shanghai, China).

### 2.2. Preparation of Colloidal Solid Dispersion Systems by Hot-Melt-Extrudate (HME)

Raw mulberry (MUL) was treated with HME (STS-25HS, co-rotating intermeshing type twin-screw extruder, Pyeongtaek, Republic of Korea) in the following way. HME used a twin-screw extruder and had a die; 1 mm, raw material injection speed; 40 g/min, 150 rpm; temperature, 70~100 °C. The extrudate after the extrusion molding was dried using a freeze dryer, and then obtained in powder form through a pulverizer. The composition of the various formulations is as follows: MUL-HME-F1 (mulberry powder 50%, whey protein isolate 40%, lecithin 2.5%, ascorbyl palmitate 2.5%, mannitol 5%, sodium alginate 2.5%, poloxamer 188 2.5%); MUL-HME-F2 (mulberry powder 50%, ascorbyl palmitate 5%, mannitol 35%, sodium alginate 5%, poloxamer 188 5%); MUL-HME-F3 (mulberry powder 40%, whey protein isolate 40%, lecithin 2.5%, ascorbyl palmitate 5 %, mannitol 5%, sodium alginate 5%, poloxamer 188 2.5%) (Table 1).

### 2.3. Total Flavonoid Content Analysis of MUL and HME-DDS (Hot-Melt Extrusion Drug Delivery System)

Total flavonoid content was measured according to the analysis method of Do et al. [21]. After adding 1 mL of 10% aluminum chloride hexahydrate and 0.1 mL of 0.1 mM potassium acetate to the extract, it reacted in the dark for 30 min, and absorbance was measured at 415 nm using a spectrophotometer. A standard curve was prepared using quercetin as a standard material.

### 2.4. Total Phenol Content Analysis of HME-DDS

Total phenol content was measured according to the analysis method of Lim et al. [22]. 200 uL of the extract, 1 mL of Folin-Denis, 0.8 m of sodium carbonate were added and reacted in the dark for 45 min, and then measured using a spectrophotometer. A standard curve was prepared using gallic acid as a standard material.

### 2.5. High Pressure Liquid Chromatography (HPLC) Analysis of Different MUL Formulations

HPLC analysis was performed using Agilent technologies 1200 series. Triart C18 (5 um, 250 × 4.6 mm, YMC, Kyoto, Japan) was used for HPLC column, and kuromanin chloride (cyanidin-3-glucoside) and keracyanin chloride (cyanidin-3-rutinoside) were used as standards. Flow rate, 1.0 mL/min; injection volume, 10 uL; oven temperature, 30 °C; detector wavelength, 535 nm. Mobile phase: gradient, solvent A, water: formic acid (90:10); solvent B, acetonitrile, methanol, water, formic acid (22.5: 22.5: 40: 10). Elution time (min): 0 min—A 93%, B 7%; 35 min—A 75%, B 25%; 45 min—A 35%, B 65%; 46 min—A 0%: B 100%; 50 min—A 35%, B 65%; 60 min—A 75%, B 25%; 70 min—A 93%, B 7%; 75 min—A 93%, B 7%.

### 2.6. Measurement of Particle Size, Zeta Size, and Polydispersity Index

The particle size, polydispersity index (PDI), and zeta potentialsize of HME-DDS were measured through DLS (Zetasizer Nano ZS, Malvern Instruments, Malvern, UK) using katsuhiro’s analysis method [23]. To measure DLS, the sample was dispersed in distilled water and measured.

### 2.7. Fourier-Transform Infrared Spectroscopy (FT-IR) Analysis

To confirm the molecular structure of the sample, FT-IR (FT-IR spectrophotometer and Microscope, iN 10/iS50, Thermo Scientific, Waltham, MA, USA) was used in the same way as for Chaitanya’s analysis method [24]. FT-IR measurements confirmed the formation of chemical bonds/entities.

### 2.8. SEM/TEM Instrumentation

Like Huseynov’s method [25], SEM and TEM were taken to visually confirm the particles in the sample. To visually confirm the shape of the sample, it was photographed with a field emission scanning electron microscope (S-4800, Hitachi Ltd., Chiyoda City, Japan). The samples were coated with platinum and photographed at a voltage of 5 kV. In order to visually confirm the transmittance of the sample, a TEM (field emission transmission electron microscope, JEM-2100F, JEOL, Akishima, Japan) image was taken. The sample was dispersed in methanol, dried on filter papers (1002-090, 90 mm, Whatman, Maidstone, UK), and then photographed.

### 2.9. In Vitro Release Study

To proceed with the in vitro release study of the sample, it was performed based on the method of Jin et al. [26]. At 0.5, 1, 2, 4, 8, 12, and 24 h, 2 mL of the release solution was collected for use in the analysis, and replaced with new medium of the same volume.

### 2.10. Bacterial Strains and Growth Conditions

Probiotic bacterial strains used in this study were *L. rhamnosus* and *P. pentosaceus*. They were cultured at 37 °C on MRS liquid or MRS agar medium. Pathogenic bacteria used were *S. aureus*, *E. coli*, and *E. faecalis*. They were cultured at 37 °C in BHI liquid or BHI agar medium. 

### 2.11. Confirmation of Growth Characteristics of Probiotics and Pathogenic Bacteria

The growth pattern of the microorganism used, the absorbance value according to the corresponding growth pattern, and the number of colonies at the absorbance were calculated. Probiotic strains (*L. rhamnosus*, *P. pentosaceus*) and pathogenic bacteria (*S. aureus*, *E. faecalis*, *E. coli*) were cultured in a nutrient medium, kept refrigerated as colonies, inoculated into sterile broth, and cultured for 48 h at 37 °C. The absorbance was measured at an OD value of 600 nm using UV/VIS at 2 h intervals. The colony count samples were spread onto an agar medium, cultured at 37 °C, and the number of colonies formed was measured. For probiotic strains, MRS liquid or solid medium was used. For pathogenic bacteria, BHI liquid or solid medium was used.

### 2.12. Measurement of Antibacterial Activity against Pathogenic Bacteria of HME-DDS Preparation Extract of Mulberry

To confirm the visible growth inhibitory effect of the HME-DDS preparation HME-MUL-F2 of mulberry on pathogenic bacteria, different concentrations of HME-MUL-F2 extracts were used, targeting *S. aureus*, *E. faecalis*, and *E. coli*. After treatment, the growth rate of pathogenic bacteria was measured over time and compared with that after treatment by MUL, a non-extrusion molded product. The concentration of pathogenic bacteria was fixed at 10^8^ CFU/mL and inoculated into BHI broth. The extract of HME-MUL-F2, an HME-DDS preparation, or MUL, a non-extrusion product, was then added at a concentration ranging from 1 mg/mL to 6 mg/mL. After incubating at 37 °C for 24 h, OD_600_ values were determined using UV/VIS. The growth of pathogenic bacteria was monitored over time. Those inoculated with pathogenic bacteria without adding samples were used as controls.

### 2.13. Comparison of Antibacterial Activity of MUL and HME-MUL-F2 with Probiotics

After treatment with HME-MUL-F2 and MUL extracts, *L. rhamnosus*, *P. pentosaceus* antibacterial activity was measured according to the paper disc diffusion method [27]. Pathogenic bacteria (*S. aureus*, *E. faecalis*, *E. coli*) were evenly spread onto Petri dishes with a culture medium adjusted to a final concentration of 10^6^ cfu/100 uL. The final cell density was adjusted to 10^7^ CFU 100 uL of probiotics suspension. HME-DDS formulation HME-MUL-F2 was adjusted to have a final treatment concentration of 2 mg/mL, and MUL, a non-extrusion molded product sample, were added dropwise onto a round paper disc with a diameter of 10 mm. After incubating the petri dish treated with pathogenic bacteria and samples at 37 °C for 24 h, the diameter (clear zone, mm) of the growth-inhibiting zone formed around each paper disc was measured to evaluate the antibacterial ability.

### 2.14. Proliferative Effect of Probiotics of HME-DDS Preparation Extract of Mulberry

In order to confirm the effect of the HME-DDS formulation on the growth of probiotics, *L. rhamnosus* and *P. pentosaceus* were treated with HME-MUL-F2 and the degree of proliferation was evaluated by a flat colony counting method [28].

After 50 uL of probiotics suspension was adjusted to a final cell density of 10^7^ CFU of two probiotics and HME-DDS formulation HME-MUL-F2 and non-extrusion was adjusted to a final treatment concentration of 2 mg/mL 50 uL of MUL extract, a molded product sample, they were mixed, spread onto MRS agar medium, and cultured at 37 °C. for 24 h. After 24 h of incubation, the number of colonies formed in the MRS agar medium cultured with two probiotics was counted. Probiotics cultured in MRS without sample treatment were used as a control.

### 2.15. Effect of Mulberry’s HME-DDS Formulation on pH Change of Probiotics Culture Medium

In order to check whether the application of the HME-DDS formulation could increase the release of antibacterial factors, such as phenolic acid or lactic acid by the conversion of ACN supplied as a substrate along with promoting the growth of probiotics strains, the pH of the probiotics culture medium was determined [29]. First, 1 mL of probiotics solution at a level of 10^7^ CFU/mL was inoculated into new MRS (100 mL). Then HME-MUL-F2 and MUL samples were added. The pH change of the cultured strain suspension was observed while incubating at 37 °C for 24 h. Measurements were made every 4 h using a pH meter. 

### 2.16. ACN Release Characteristics of the Added HME-MUL-F2 Formulation in Terms of the Growth of Probiotic Strains

In the medium in which probiotics strains were cultured, HME-MUL-F2 treatment resulted in lower pH decrease than when the treatment was with the non-extrusion product MUL, due to continuous release of MUL as a characteristic of the HME-DDS formulation. Contents of ACN distributed in the culture medium of probiotics were tested each hour after HME-MUL-F2 treatment and release characteristics of HME-MUL-F2 were confirmed. For probiotics culture and sample processing, the same method used for the pH change assay was applied. The content of ACN in probiotics culture was analyzed by HPLC. After processing each sample and co-culturing, 3 mL of HPLC analysis sample was collected every 4 h and replaced with new medium of the same volume. The obtained HPLC sample was filtered through a 0.45 um syringe filter and the amount of ACN was measured. The percentage of cumulative release at each time was determined by testing the content of cyanidin-3-glucoside (C3G) and cyanidin-3-rutinoside (C3R) contained in the HPLC sample using MUL and HME-MUL-F2 obtained at each h. The release rate was calculated as a relative ratio of amounts of C3G and C3R.

### 2.17. Effect of HME-MUL-F2 Formulation on Antibacterial Activity of Probiotics

Next, the treatment effect of HME-MUL-F2 on the antibacterial effect of the probiotic strain on pathogenic bacteria was investigated. The antibacterial activity exhibited by additional combination treatment with HME-MUL-F2 of mulberry in non-extrusion (MUL) and probiotics cultures alone, *L. rhamnosus*, and *P. pentosaceus* was confirmed by the paper disc diffusion method. First, the culture medium adjusted to the final concentration of 10^6^ cfu/100 uL of pathogenic bacteria (*S. aureus*, *E. faecalis*, *E. coli*) was spread evenly onto a petri dish containing BHI agar medium. The diameter was 10 mm inside the medium on which pathogenic bacteria were smeared. 100 uL of cell suspensions of *L. rhamnosus* and *P. pentosaceus* were adjusted to HME-MUL-F2 2.8 mg, MUL 6.7 mg, and a final cell density of 10^7^ CFU by punching 4 round wells. Finally, *L. rhamnosus*, 100 uL of *P. pentosaceus* cell suspension, and 2.8 mg of HME-MUL-F2 were combined and used for treatment. After the pathogenic bacteria were smeared and the sample-treated petri dish was cultured at 37 °C. for 24 h, the diameter (clear zone, mm) of the growth-inhibiting zone formed around each well was measured to evaluate the antibacterial ability.

### 2.18. Statistical Analysis

Results are expressed as means. For statistical programs, SPSS software (IBM, Armonk, NY, USA) was used. All analyses were analyzed between groups through one-way ANOVA with *p* < 0.05 level. The results were verified through Tukey’s Range Test.

## 3. Results

### 3.1. Total Flavonoids, Phenol Contents, and Anthocyanin Contents of MUL and HME-DDS

The HME process could increase the content of polyphenols by hydrolyzing fibers or proteins, positively affecting total phenol content [30,31]. High-extrusion temperature can cause decomposition or structural changes of phenol compounds [32]. Therefore, phenolic compounds might be weakly chemically reactive or more difficult to extract due to their specific degree of polymerization [33]. HME can maximize total phenol contents of chemical-extruded products. When comparing phenol contents, samples extruded with only the raw material (MUL-HME) and those extruded with the polymer (MUL-HME-F1, MUL-HME-F2, MUL-HME-F3) were compared to samples of the raw material itself (MUL). It was confirmed that the content of total phenols in MUL-HME-F3 was the highest.

Heat and pressure are applied to most fruits and vegetables, and hydrolysis of flavonoid glycosidic bonds can occur through this process to form monomers [34]. Additionally, in most fruits and vegetables, flavonoids form glycoside bonds and exist as dimers and oligomers. Industrial processes such as heating can lead to formation of monomers by hydrolysis of glycoside bonds [34]. Total flavonoids contents in the sample of the raw material itself (MUL) and the sample extruded with only the raw material (MUL-HME) were also compared. It was found that samples extruded with the polymer (MUL-HME-F1, MUL-HME-F2, MUL-HME-F3) had high contents of total flavonoids (Table 2).

**Table 2:** Total phenol contents, total flavonoid contents, and total ACN contents in MUL extracts.

		Total Phenol Contents (mg/g)	Total Flavonoid Contents (mg/g)
Raw materials	MUL	7.19 ± 3.14 ^e^	3.50 ± 0.20 ^f^
	MUL-CA	9.26 ± 1.17 ^e^	5.29 ± 0.57 ^de^
	MUL-F1	28.07 ± 3.06 ^c^	8.19 ± 2.09 ^b^
	MUL-F2	25.64 ± 0.77 ^cd^	6.60 ± 0.88 ^cd^
	MUL-F3	26.99 ± 0.52 ^cd^	7.75 ± 0.58 ^bc^
Extrusion materials	HME-MUL	6.82 ± 2.28 ^e^	3.13 ± 0.09 ^f^
	HME-MUL-CA	21.97 ± 0.96 ^d^	4.95 ± 0.88 ^e^
	HME-MUL-F1	37.71 ± 7.04 ^b^	8.33 ± 0.26 ^b^
	HME-MUL-F2	29.90 ± 0.99 ^c^	9.88 ± 0.98 ^a^
	HME-MUL-F3	46.71 ± 0.86 ^a^	6.84 ± 0.31 ^bc^

MUL: mulberry; MUL-CA: MUL treated with 0.5 M citric acid; MUL-HME: only solid formulations of the extrudate of MUL; MUL-HME-CA: solid formulations of the extrudate of MUL treated with 0.5 M citric acid. HME-MUL-F1/F2/F3: treatment of mulberry HME-DDS formulation. ^a,b,c,d,e,f^ values differences in the same condition (*p* < 0.05). Data are presented as mean ± SD (*n* = 3). Since HME can increase the reactive surface area of a compound and destroy the fiber matrix, it is known as the most appropriate cell wall structure destruction method for active compound extraction [35]. After comparing ACN content with total ACN content, the samples extruded with polymer (MUL-HME-F1, MUL-HME-F2, MUL-HME-F3) showed a higher content than the sample extruded with only the raw material (MUL-HME) (Table 3). This might be due to cell wall structure destruction by HME. In total phenol content analysis, the sample of MUL-HME-F3 had the highest content, with decreasing tendency in the order of MUL-HME F3, F1, and F2. When the contents of total flavonoids and anthocyanins were compared, it was confirmed that the contents decreased in the order of MUL-HME-F2, F1, and F3.

These results could increase the extraction efficiency of phenolic compounds and ACN by applying high-level pressure and shear force to a material using the extrusion process. Hu et al. [7] have found that extraction efficiency for phenolic compounds from the developed material is increased when an extrusion molding process is applied, due to the strong shear force applied in the extrusion molding process. They reported that the reason was because cross-linking and ester bonds were partially broken. The increase in content of phenols and flavonoids was due to an increase in extractability of bound phenols by pyrolysis of cellular components [39]. This is similar to a previous report showing that the bioavailability of phenolic acid is increased when the particle size is reduced by mechanical treatment [40,41]. Additionally, samples prepared using the extrusion molding process showed increased contents of C3G and C3R (ACNs of mulberry) released into the solution. It is presumed that extracting ACN from mulberry raw material with water without using an organic solvent or additional conditions such as addition of acid is not sufficient. By destroying the cell wall and ACN vacuolar inclusions (AVI) that exist as a phosphorous barrier, it is believed that ACNs could be easily eluted by diffusion even with water as a solvent.

Sodium alginate is an anionic polysaccharide found as a structural component in cell walls of brown algae *Ascophyllum nodosum*, *Macrocystis pyrifera*, and *Laminaria hyperborean* [42]. It is insoluble in water and organic solvents. Alginic acid has been used as an emulsifier, thickener, formulation aid, and stabilizer. Under physiological conditions, alginate is degraded because of partial oxidation of the alginate chain. Alginate is oxidized in an aqueous medium to aid hydrolysis [43]. The glass-transition temperature (Tg) value of alginate is 110 °C. Thus, it is difficult to form amorphous polymers; a higher temperature is required for the phenomenon [43]. As the temperature of the alginate solution increases, the viscosity decreases. Thermal decomposition occurs more rapidly at higher temperatures and lower pH values. Among HME-DDS formulations of mulberry, MUL-HME-F3 showed a relatively low extraction yield. During extrusion, the gel was processed at a low temperature with an acid treatment and a low pH environment for stable production of ACNs.

### 3.2. Characterizations of MUL and MUL-DDS Formulations

A way to improve drug solubility is to reduce the size of drug particles to a nanometer scale, which can greatly increase specific surface area (area per mass). According to the Noyes–Whitney equation, the decrease in particle size can increase the drug particle dissolution rate [44]. Reducing particle size can also induce surface curvature at the liquid/displacement interface, which can increase dissolution pressure and drug solubility regarding vapor pressure between liquid and gas in Kelvin and Ostwald–Freundlich equations [45].

One study has reported that an extruder (HME; hot melt extrusion) can increase the pressure acting on the mass to make uniform thickness, shape, and size [46]. By measuring the particle size of each sample with a particle size analyzer, the particle size of the extruded sample (MUL-HME-F1, MUL-HME-F2, MUL-HME-F3) with polymer was found to be smaller than that of the mulberry raw material itself (MUL). The particle size was 329.67 ± 13.37 nm for mulberry raw material itself (MUL), 258.63 ± 21.73 nm for MUL-HME-F1, 152.03 ± 3.19 nm for MUL-HME-F2, and 218.20 ± 61.48 nm for MUL-HME-F3 (Table 4). These results confirmed that the particle size decreased during the HME process. Lee et al. [47] have stated that HME is the most suitable process for amorphizing crystalline materials by reducing particle size and improving solubility [48]. Therefore, for extruded mulberry samples, useful components can be amorphized by reducing particle size to constitute a solid dispersion. In addition, the solubility can be improved and the bioavailability can be increased.

Particle size plays an important role in the GI absorption of nanoparticles after oral administration. A particle size of less than 300 nm is suitable for effective intestinal transport. As a result of the comparative analysis of zeta potential and PDI, the surface charges of all samples were found to be negative. In the case of MUL-HME-F2, the absolute value was more than 30. More negative surface charge values formed a surface charge greater than an absolute value of 30 because the molecular weight of poloxamer 188 and the hydrophilicity/hydrophobicity ratio of functional groups affected size and stability [49]. PDI values were less than 0.300 for MUL-HME-F2 and MUL-HME-F3 samples. As reported previously, ACN particles using extrusion are uniformly distributed. They can be used for long-term transport.

### 3.3. Structural Change of Compounds MUL and HME-DDS Using FT-IR

**Figure 1:**
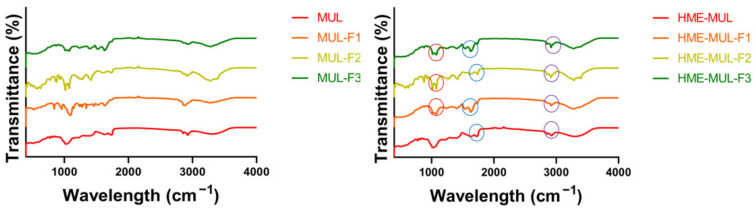
FT-IR analysis of the MUL extrudate’s solid formulation obtained using different chemical additives.

FT-IR is the most suitable spectroscopic method for the non-destructive analysis of samples because it can identify functional groups by confirming their absorbance and transmittance [50]. As a result of comparing FT-IR spectra of extruded samples (MUL-HME-F1, MUL-HME-F2, MUL-HME-F3) with those of the polymer and the non-extruded mulberry sample (MUL), the sample MUL did not have a peak in the range of 1700–3500 cm^−1^, whereas samples extruded with a polymer (MUL-HME-F1, MUL-HME-F2, MUL-HME-F3) had several split peaks in the range of 1700–3500 cm^−1^, indicating that a new functional group was formed (Figure 1). This functional group was found to have high peaks in the order of MUL-HME-F2 > MUL-HME-F1 > MUL-HME-F3. The peaks at 1700–1500 cm^−1^ have the characteristics of a methyl group, and the peaks at 3500 and 3000 cm^−1^ are related to C-H stretching and OH stretching bands. The peak at < 2000 cm^−1^ represented a carbonyl group with = C bond in the aromatic ring and an aromatic CH bond in the substituted ring [51]. A peak of 1500 cm^−1^ or less was related to the carbon–oxygen bond (CO) of ethers, esters, and carboxylic acids present in various metabolites, such as tannin, flavonoid, and anthraquinone [52].

Comparing the spectra of a non-extruded sample and an extruded sample, it was confirmed that a peak at the wavelength of 1700 cm^−1^ was newly generated in the extruded sample. The peak at the wavelength of 1700 cm^−1^ indicated the C=O bond. After the extrusion process, it was confirmed that a carboxyl group (COOH) stretching band was newly formed. These results are similar to those of a previous report showing that the carboxyl group in the structural change of the compound is closely related to the water solubility of the agent. The formation of a split peak in the wavelength band of 1700 cm^−1^ indicates an increase in the carboxyl group. It is known that an increase in the carboxyl group can increase the solubility of an agent [53]. From the above results, it was confirmed that the water solubility of the formulation containing mulberry was increased after the application of extrusion molding technology. Therefore, MUL-HME-F2 with its high contents of total flavonoids and ACNs, the lowest PSA, a PDI of 0.3 or less, and a ZP of over 30 mV was used for the next experiment.

### 3.4. Confirmation of Particle Surface Morphologyof MUL and HME-DDS

**Figure 2:**
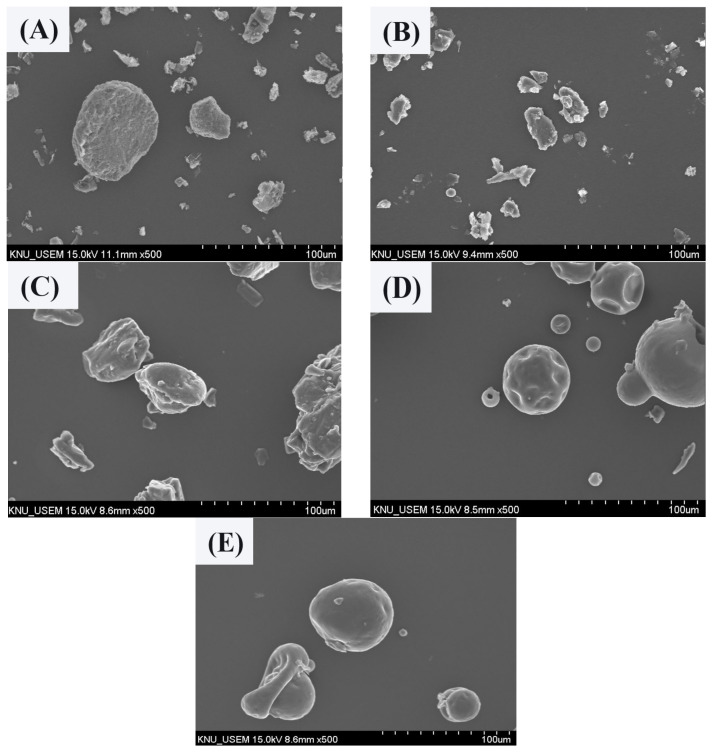
Scanning electron microscopy images of MUL and HME-DDS (×500). (**A**) MUL powder (MUL), (**B**) only MUL powder extrudate (MUL-HME), (**C**) MUL-HME-F1, (**D**) MUL-HME-F2, (**E**) MUL-HME-F3.

**Figure 3:**
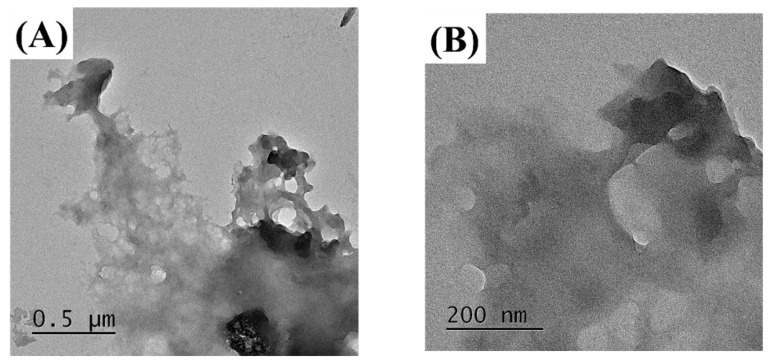

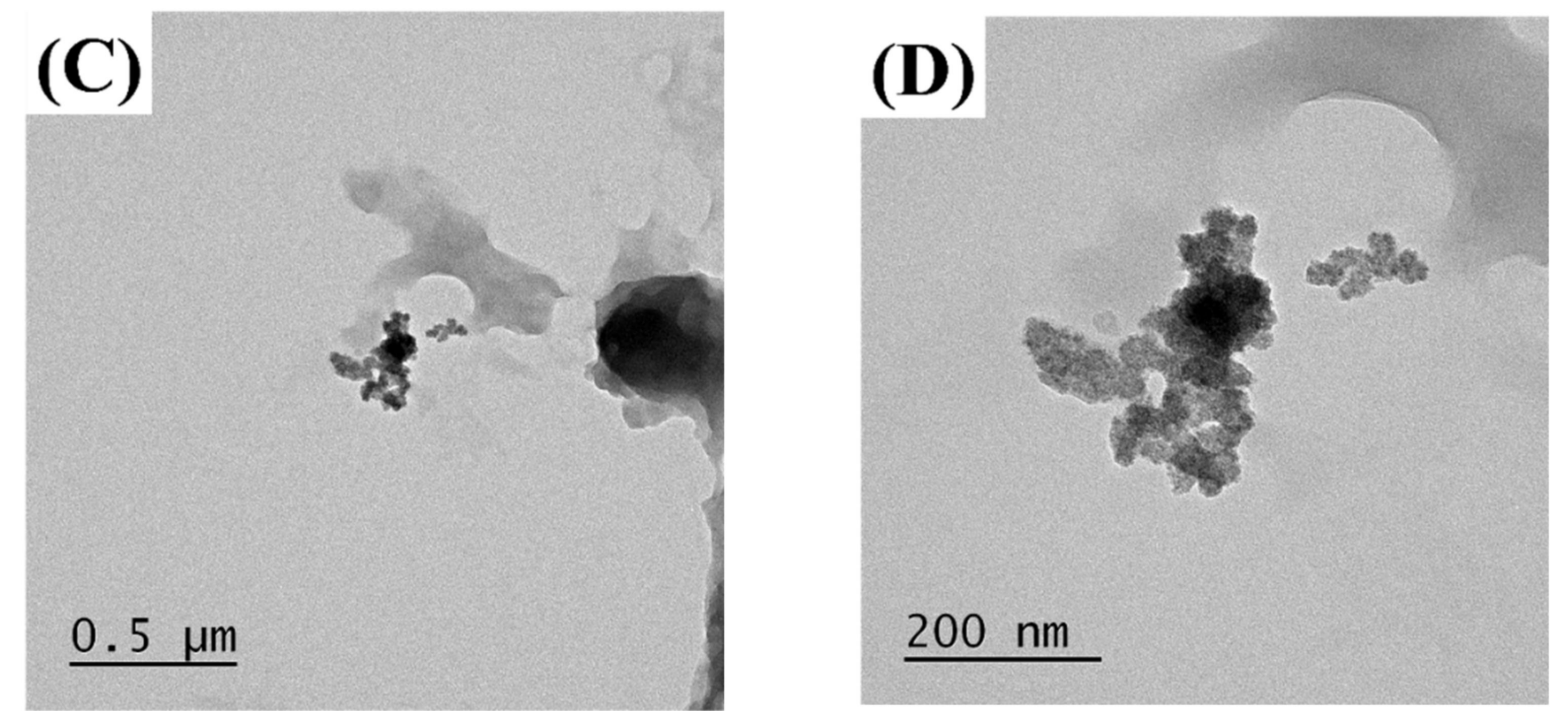
Transmission electron microscopy images of MUL and HME-DDS. (**A**,**B**), MUL powder (MUL), (500 nm, 200 nm), (**C**,**D**) MUL-HME-F2 (500 nm, 200 nm).

The MUL and MUL-HME-F2 were visualized at ×1000 and ×5000 magnifications using a scanning electron microscope (SEM) (Figure 2). It was observed that the surface of the MUL was relatively rough and the size varied between particles. However, the surface of the sample MUL-HME-F2 was generally smooth and the intergranular size was regular. Moreover, when using 5000 × magnification, it was confirmed that the surface of particles was smooth and voids on the surface of particles were reduced (Figure 3). Therefore, it was judged that the uniformity of particles could be improved by HME process. Thus, the reproducibility and uniformity of intestinal release could be increased. Nanoparticles provide sustained release that can reduce dosing frequency and increase patient compliance [54].

### 3.5. In Vitro Release of non-HME, MUL, and MUL-DDS

**Figure 4:**
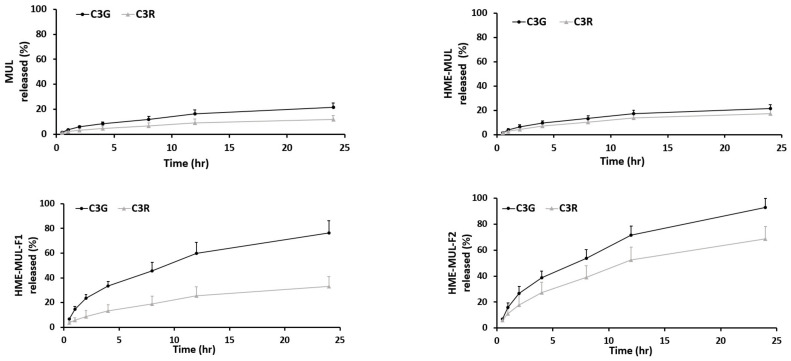

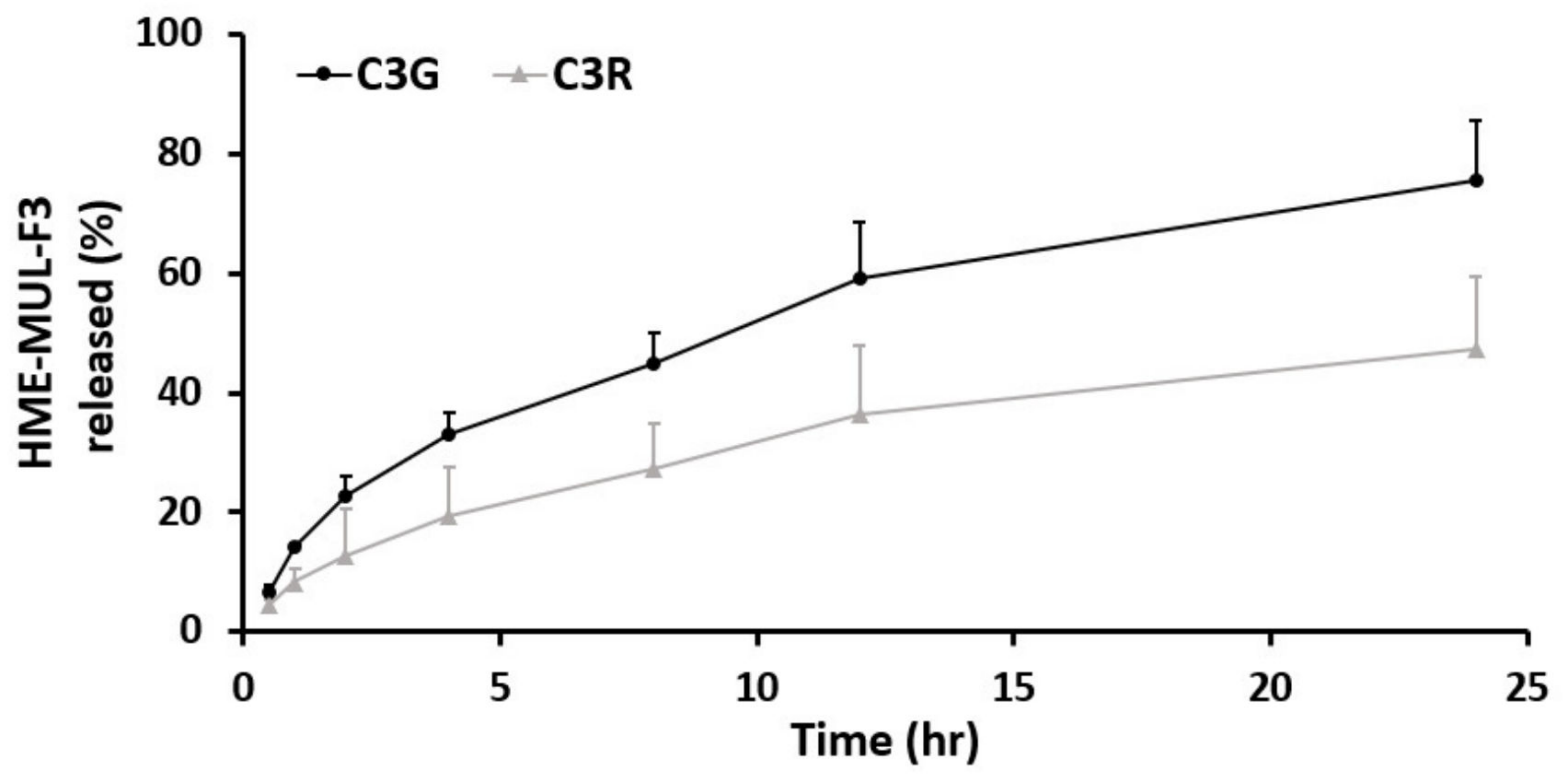
Release profile of ACN (C3G and C3R) from MUL, HME-MUL and HME-DDS (HME-MULF1, F2, F3) in gastrointestinal tract simulation. SGF: simulated gastric fluid, from initial and 2 h (0, 0.5, 1, and 2 h), SIF: simulated intestinal fluid from 2 to 24 h (2, 4, 8, 12, and 24 h).

In vitro release studies of MUL and MUL-HME-F2 were carried out in the artificial stomach and small intestine. After checking emission characteristics of extruded products (MUL-HME-F1, F2, and F3) containing mulberry raw material itself (MUL) and excipients, low emission characteristics were found as follows: MUL, C3G: 21.38%, C3R: 11.99%; MUL-HME-F1, C3G: 76.19%, C3R: 33.03%; MUL-HME-F2, C3G: 92.85%, C3R: 68.66%; MUL-HME-F3, C3G: 75.52%, C3R: 47.26% (Figure S1, Figure 4). It was confirmed that MUL-HME-F2 exhibited the best emission characteristics. It was thought that ACN was stably released in the form of flavylium in an acidic environment (pH 1–3) in the stomach. Decomposition at pH 7 [55] was confirmed in this release characteristic. The sample of MUL-HME-F2 extruded with polymer showed significantly increased release compared to the raw material of mulberry itself (MUL). Its release was significantly higher than that of extruded sample treated with alginate or CA, but lower than that of the artificial gastrointestinal environment. Release characteristics were consistent with previous reports showing that the diffusion distance decreased whereas the dissolution rate increased after the addition of excipients [56].

During the HME process, the biopolymer is depolymerized due to heat and high shear stress [57,58]. In addition, the drug and the active compound become soluble. According to the above results, when mulberry sample was mixed with acid, ascorbyl palmitate, mannitol, poloxamer, or sodium alginate under the extrusion process, the chain of the alginate polymer was decomposed due to temperature and shear stress. ACNs of mulberry, whose molecular structure has been changed, are dispersed and placed in the polymer matrix. Again, as the temperature becomes lower, ACNs are trapped into mannitol and sodium alginate to form an extrudate, exhibiting high release properties.

Poloxamer 188 could increase intracellular uptake of molecules, such as sugars, drugs, proteins, and DNA by forming pores in the cell membrane [59]. The polymer mixed on the particle surface by penetrating the nasal mucus can improve transport of the mucus and increase absorption of particles [60]. Coating particles with starch, sodium alginate, or protein base generally protects the active ingredient from being exposed to an acidic environment of the stomach, thus preventing hydrolysis and allowing release in the small intestine [61]. 

### 3.6. Confirmation of Probiotics and Pathogenic Bacteria Growth Characteristics

The growth pattern of general bacterial culture consists of a delayed phase → logarithmic or exponential growth phase → deceleration phase → stationary phase → death phase. Each phase represents the reaction process of different cells. Growth characteristics of lactic acid bacteria (LAB) and pathogenic bacteria were confirmed by measuring the optical density and the number of colonies at the corresponding OD value. Because of confirming numerically, it took about 4–8 h for LAB and 16 h for pathogens. In the case of logarithmic growth, when the mass and number of cells increased exponentially, LAB was distributed from 8 h to 28 h to 30 h (Figure S2). For pathogens, they were distributed from 18 h to 24 (up to 34) h for *S. aureus*, *E. faecalis*, and *E. coli*.

### 3.7. Determination of Antibacterial Activity against Pathogenic Bacteria of HME-DDS Formulation Extract of Mulberry

**Figure 5:**
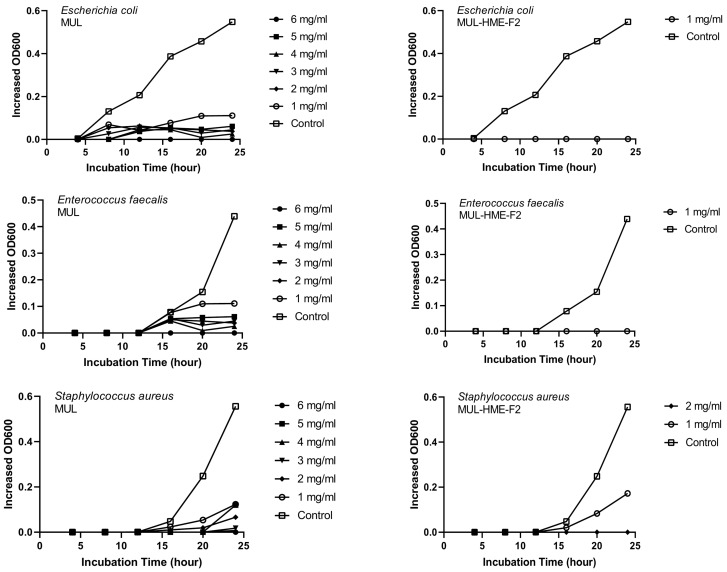
Growth inhibition effects of mulberry raw materials and HME-DDS formulations (HME-MUL-F2) on pathogen bacteria.In the case of HME-MUL-F2, bacterial growth was completely inhibited at a relatively lower treatment concentration. Since HME-MUL-F2 was composed of a highly water-soluble ascorbyl palmitate as an excipient, it could inhibit the growth of bacteria or other microorganisms [62]. As for the mannitol, it could enhance the thermal stability of the mixed material. The poloxamer 188 and sodium alginate played a role in protecting the active ingredient. As reported previously, ACNs can inhibit the growth of *E. coli* and *S. aureus* [63]. HME-MUL-F2 showed antibacterial activities even at low concentrations. It is confirmed that the antibacterial effect on higher pathogens was exhibited under the influence of the excipients used.

MUL and HME-MUL-F2 were treated with *S. aureus* and *E. faecalis*. In the case of *E. coli*, the antibacterial effect was confirmed after 16 h, which is the aquatic growth phase. In the case of MUL, as the treatment concentration increased, antibacterial effects on all three pathogenic bacteria also increased dependent on concentration. When the highest concentration (6 mg/mL) was used, a complete growth inhibitory effect was detected (Figure 5).

### 3.8. Effect of MUL and HME-MUL-F2 on the Antibacterial Ability of Probiotics

**Figure 6:**
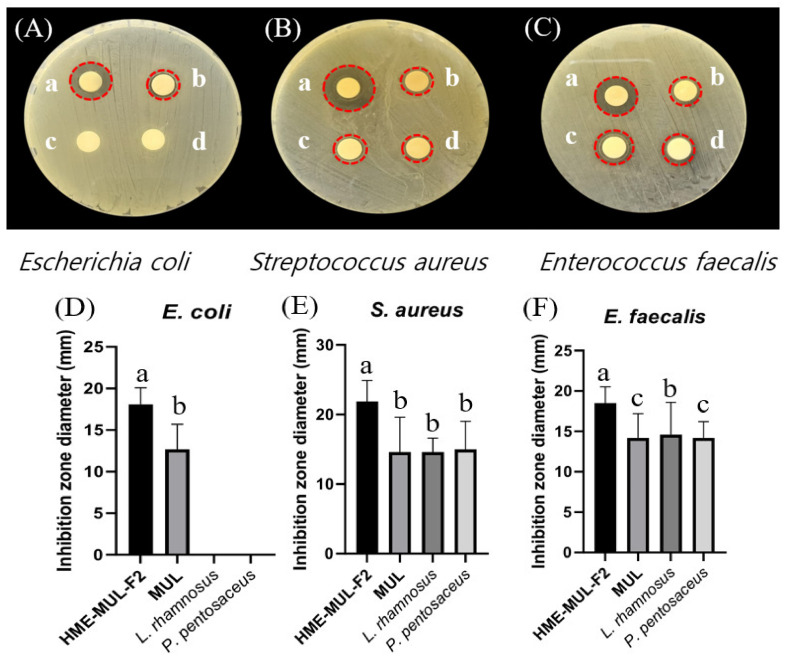
Inhibition zones in the presence of probiotics (*L. rhamnosus* and *P. pentosaceus*), mulberry raw materials (MUL), and HME-DDS formulation (HME-MUL-F2). (**A**,**D**): inhibition zone of *E. coli*. (**B**,**E**): inhibition zone of *S. aureus*. (**C**,**F**): inhibition zone of *E. faecalis*. a: treatment with HME-MUL-F2; b: treatment with MUL; c: treatment with *L. rhamnosus*; d: treatment with *P. pentosaceus*. (**A**–**C**): photo of clear zone after treatment with probiotics, HME-MUL-F2, and MUL. (**D**–**F**): quantification of inhibition zone diameter after treatment with probiotics, HME-MUL-F2, or MUL. a,b value differences in the same condition (*p* < 0.05). Data are shown as mean ± SD (n = 3).

The antibacterial activities of MUL and HME-MUL-F2 with or without probiotics *L. rhamnosus* and *P. pentosaceus* were determined using the paper disc diffusion method (Figure 6). With respect to the pathogen *E. coli*, probiotics (*L. rhmanosus* and *P. pentosaceus*) did not form a growth inhibitory ring, indicating that the probiotics did not show antibacterial activities against *E. coli*. However, after the treatment with MUL or HME-MUL-F2, the diameter of the growth inhibition ring was 12.7 ± 0.3 mm or 18.1 ± 0.2 mm, respectively, confirming that MUL and HME-MUL-F2 had antibacterial effects on *E. coli*. For pathogen *S. aureus*, the diameter of the growth inhibition ring was 14.6 ± 0.7 mm after the treatment with MUL, while 21.9 ± 2.2 mm was observed after the treatment with HME-MUL-F2. In addition, 14.7 ± 1.1 mm and 15.0 ± 0.3 mm was measured after the treatment with probiotics *L. rhamnosus* and *P. pentosaceus*. These results confirmed the treatments’ antibacterial effects on *S. aureus*. For pathogen *E. faecalis*, the diameter of the growth inhibition ring was 18.5 ± 0.2 mm after treatment with MUL, 14.2 ± 0.3 mm after treatment with HME-MUL-F2, 14.6 ± 0.4 mm after treatment with *L. rhamnosus*, and 14.2 ± 0.2 mm after treatment with *P. pentosaceus*. These results confirmed their antibacterial effects against the pathogen *E. faecalis*. HME-MUL-F2 showed higher antibacterial activity than raw material extracts or probiotics at the same concentration against all pathogens. ACNs have an antibacterial effect by inhibiting the growth of pathogens, such as *E. coli* and *S. aureus* [64]. ACNs and their metabolites are permeable to bacterial cells. Thus, a large amount of the matrix material inside the bacterial cell can be leaked out, exhibiting an antibacterial effect [65].

### 3.9. Effects of Sterilized MUL and HME-MUL-F2 on Growth of Probiotics

**Figure 7:**
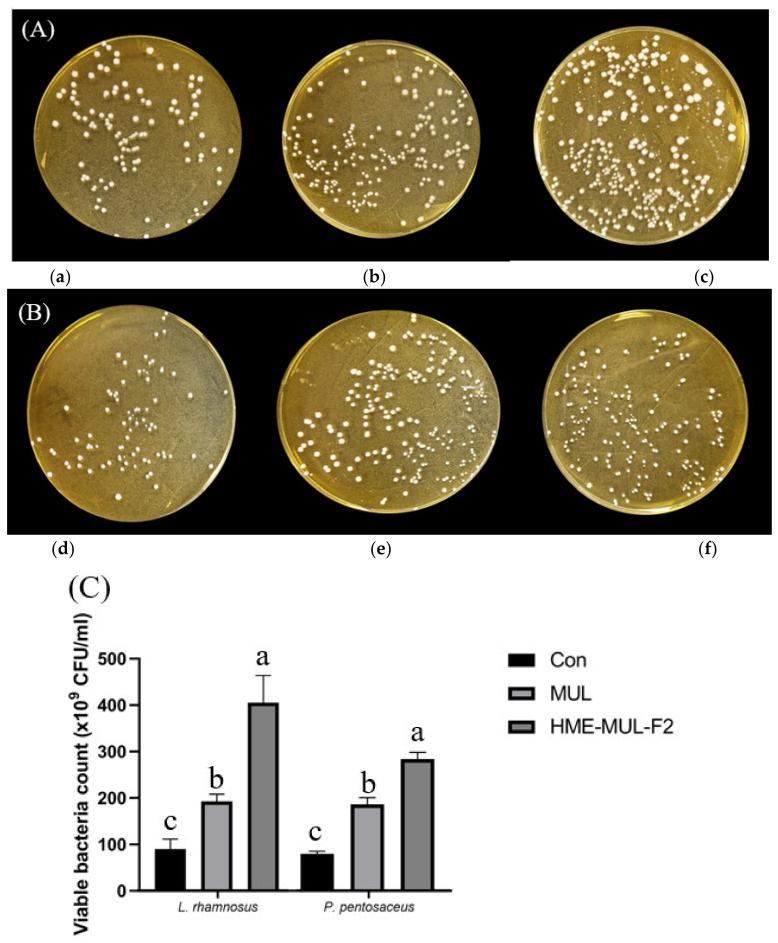
Photos of probiotics colonies co-cultured with mulberry raw materials (MUL) and HME-DDS formulation (HME-MUL-F2). (**A**) *L. rhamnosus*, (**B**) *P. pentosaceus*, (**C**) Quantification of probiotics colonies in the presence of MUL and HME-MUL-F2. (**a**) *L. rhamnosus*, (**b**) L. rhamnosus + MUL (2 mg/mL), (**c**) *L. rhamnosus* + HME-MUL-F2 (2 mg/mL), (**d**) *P. pentosaceus*, (**e**) *P. pentosaceus* + MUL (2 mg/mL), (**f**) *P. pentosaceus* + HME-MUL-F2 (2 mg/mL). a,b,c value differences in the same condition (*p* < 0.05).Data are presented as mean ± SD (n = 3).

To investigate the effects of HME-MUL-F2 on the growth and division of LAB probiotic cells, the degree of growth and division of LAB probiotic cells were determined. After calculating the number of colonies, it was confirmed that the number of colonies formed in the presence of both non-extrusion molded product MUL and HME-DDS formulations was increased. HME-DDS preparation and HME-MUL-F2 increased the number of LAB cell colonies more than non-extrusion MUL. HME-MUL-F2 showed a colony number of 405.67 × 10^9^ CFU/mL, confirming that it exhibited a proliferation-promoting effect on *L. rhamnosus* by 4 times or more compared to the control (*L. rhamnosus* culture only without treatment). In the case of *P. pentosaceus*, the control group showed 75.00 × 10^9^ CFU/mL within 24 h of incubation (Figure 7). After treatment with non-extruded product MUL, it exhibited 175.00 × 10^9^ CFU/mL, and showed a 2.3-fold increase in proliferation. HME-MUL-F2 promoted its proliferation by 3.56-fold to 267.00 × 10^9^ CFU/mL. These results indicate that the HME-DDS preparation HME-MUL-F2 can be effectively used for the growth of lactic acid bacteria. It is thought that it can be effectively used to maintain the balance of intestinal microflora.

### 3.10. Effect of HME-DDS Formulation of Mulberry on pH Change of Probiotics Culture Medium

In the lactic acid bacterial culture and fermentation process, a decrease in medium pH indicates the production of organic acids containing phenolic acid and short-chain fatty acids. Supplied ACNs can be used as a substrate of lactic acid bacteria and converted into metabolites exhibiting antibacterial activities. It has been reported that ACNs can be used by human intestinal bacteria [66]. Additionally, C3G, the main ACN of mulberry, represents the first step in numerous reactions, such as hydrolysis, dihydroxylation, and methoxylation. Due to the presence of β-glucosidase secreted by lactic acid bacteria, glucoside can be lost. It can be degraded to cyanidin in the form of aglycone [67]. After that, a heterocycle cleavage of cyanidin is performed by lactic acid bacteria, resulting in the existence of a chalcone pseudo base [68]. Finally, cyanidin and chalcone are decomposed through methylation and present as phenolic acid. These phenolic acids and metabolites lower pH, and as the metabolites increase, the media environment becomes acidified, which can promote mineral absorption [69].

**Figure 8:**
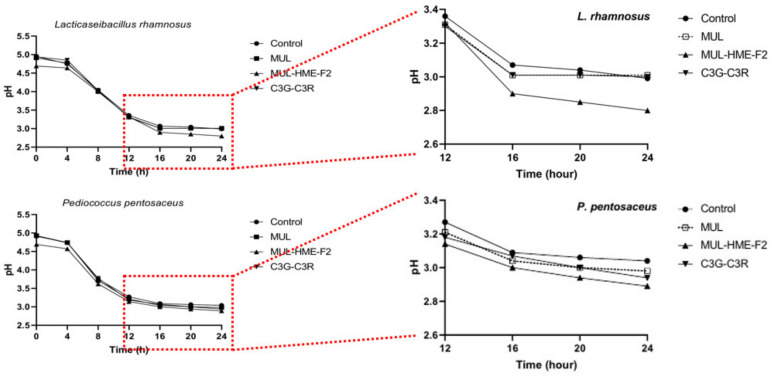
Changes of pH value for *L. rhamnosus* and *P. pentosaceus* treated with mulberry raw materials (MUL) and HME-DDS formulation (HME-MUL-F2). LAB probiotics were co-cultured with sample in MRS broth for 24 h. The decrease in pH according to the treatment with HME-MUL-F2 can be explained by the HME-DDS. The HME-DDS preparation could improve the growth and metabolism of the LAB strain. A large amount of organic acid was formed by the probiotics strain, which promoted the growth of the probiotics.

Whether treatment with HME-DDS preparation, HME-MUL-F2, could affect the formation of phenolic acid or the release of antibacterial factors such as lactic acid by the conversion reaction of ACN supplied as a substrate along with the growth promotion of LAB strains remains unclear (Figure 8). To check this, the pH change of the medium over time was measured. A low pH value was exhibited after treatment with HME-MUL-F2 and a significant decrease in pH was confirmed in both types of LAB strains after 24 h of incubation. At 24 h after incubation, the pH of *L. rhamnosus* bacteria was 2.99, MUL 3.01, HME-MUL-F2 2.80, and C3G-C3R 3.00. The pH of the 24 h culture of *P. pentosaceus* was 3.04, MUL 2.98, HME-MUL-F2 2.89, C3G-C3R 2.94.

In the case of non-extrusion moldings treated with MUL, C3G, or C3R, the overall pH was lower than that of the control, although the differences were not statistically significant. Probiotics can secrete antimicrobial molecules such as lactic acid, bacteriocins, and enzymes that can inhibit pathogenic microorganisms during growth. The molecules are thought to be able to increase their metabolic activity.

### 3.11. Anthocyanin Release Characteristics after HME-DDE Prepation of Mulberry Is Added to Probiotics Strains

**Figure 9:**
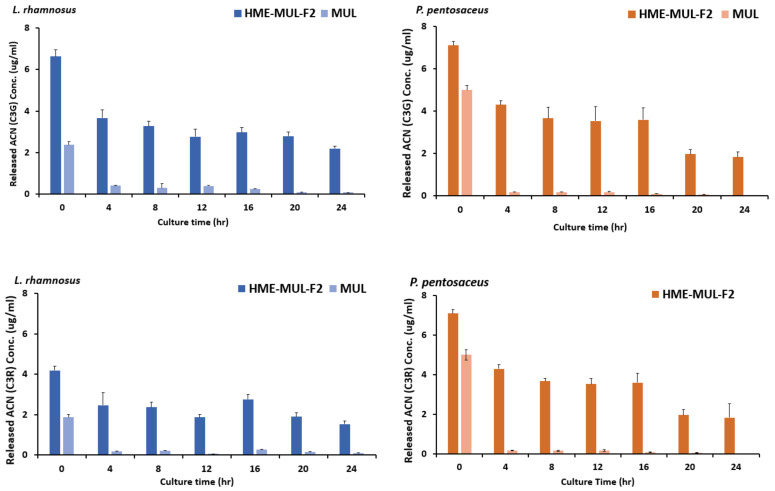

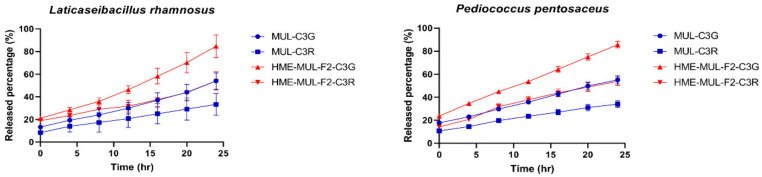
Released anthocyanins content and release profile of *L. rhamnosus* and *P. pentosaceus* cultured with mulberry raw materials (MUL) and HME-DDS formulate (HME-MUL-F2).The trend of the ACN content in *P. pentosaceus* after treatment with HME-MUL-F2 formulation was similarly observed. In the case of MUL, C3G and C3R contents were 5.0 ug/mL and 3.3 ug/mL, respectively, at the beginning of the culture. At 4 h after incubation, they were decreased to 0.17 ug/mL and 0.13 ug/mL, respectively. At 24 h after incubation, they were decreased to a level of 0.02 ug/mL. In the case of the HME-MUL-F2 formulation, C3G and C3R contents were 7.1 ug/mL and 4.3 ug/mL, respectively, at the beginning of the culture. They were decreased to 4.3 ug/mL and 1.9 ug/mL, respectively, after 4 h of incubation. In the case of C3G, it was maintained at 3.6 ug/mL until 16 h. Thereafter, it was maintained at 1.8–1.9 ug/mL until 24 h. In the case of C3R, it was maintained at a level of 1.0–1.5 ug/mL from 8 h to 24 h. As in the case of HME-DDS formulation and HME-MUL-F2, intestinal dissolution characteristics of an artificial gastrointestinal environment after treatment with HME-DDS formulation were confirmed. Although the ACN present in the HME-MUL-F2 formulation was released into the medium of *L. rhamnosus* and *P. pentosaceus* culture and converted into phenolic acid and organic acid by intestinal microbial metabolism, the content of ACN decreased. It was thought that ACN was maintained at a certain level in the medium even after incubation time elapsed due to the slow and continuous release or sustained-release nature of the formulation.

After adding the HME-DDS formulation, HME-MUL-F2, and MUL to the culture medium of LAB probiotics, the contents of ACN released into the medium were tested as the culture progressed. It was found that MUL reduced the release of ACNs in *L. rhamnosus* culture. The C3G content was 2.3 ug/mL at the beginning of the culture. It was sharply decreased to 0.40 ug/mL after 4 h of incubation with HME-MUL-F2 (Figure 9). The content of C3R was also decreased continuously from 1.87 ug/mL at the beginning of the culture to 0.10 ug/mL at 24 h after incubation with HME-MUL-F2. In the case of non-extrusion molded products (MUL), the ACN content gradually decreased when *L. rhamnosus* culture progressed. As time passed, the ACNs contained in MUL acted as a substrate and phenols went through intestinal microbial metabolism. They were thought to be converted into acids and organic acids. Thus, ACN content was reduced. When *L. rhamnosus* culture was treated with HME-DDS preparation, HME-MUL-F2, C3G content was 6.63 ug/mL and C3R content was 4.2 ug/mL at the beginning of culture. At 4 h after incubation, the C3G content was lowered to 3.6 ug/mL and the C3R content was decreased to 2.4 ug/mL. However, from 16 h to 24 h of incubation, C3G content was maintained at 2.2–2.9 ug/mL and C3R content was maintained at 1.5–1.8 ug/mL.

Additionally, the ACN present in the HME-MUL-F2 preparation existed in the form of sodium alginate and protein. It could be degraded by the action of various enzymes in the intestinal environment where the pH would change rapidly, especially in a high pH environment such as pH 5.5. It is thought that HME-DDS can act while maintaining a long half-life in each part of the gastrointestinal tract by preventing loss of ACN. As a result of confirming release characteristics, the release rate of C3G from MUL in *L. rhamnosus* increased was from 13.4% at the beginning of culture to 54.0% at 24 h and that of C3R changed from 8.4% at the beginning of culture to 33.4% at 24 h. In the case of HME-DDS formulation, the release rate of C3G was increased from 21.0% at the beginning of culture to 84.75% at 24 h. In addition, in *P. pentosaceus*, the release rate of C3G from MUL was 17.75% at the beginning of culture and 55% at 24 h of culture. In the case of the HME-DDS formulation, the release rate of C3G was increased from 23.66% at the beginning of the culture to 85.7% after 24 h and the release rate of C3R was increased from 14.33% at the beginning of the culture to 53.8% after 24 h. In the case of the MUL-F2 formulation, it was confirmed that the release rate was further increased over time in an environment in which probiotics cells were cultured.

### 3.12. Effect of HME-DDS Formulation of Mulberry on Antibacterial Activity of Probiotics

Increased probiotics and decreased pH of the medium positively correlate with the antimicrobial activity of probiotics, which can regulate intestinal microbes by competing with pathogenic bacteria and inhibiting growth [70]. It has also been reported that retardation of probiotics is beneficial for intestinal microflora, although probiotics alone do not restore intestinal microflora diversity [71].

**Figure 10:**
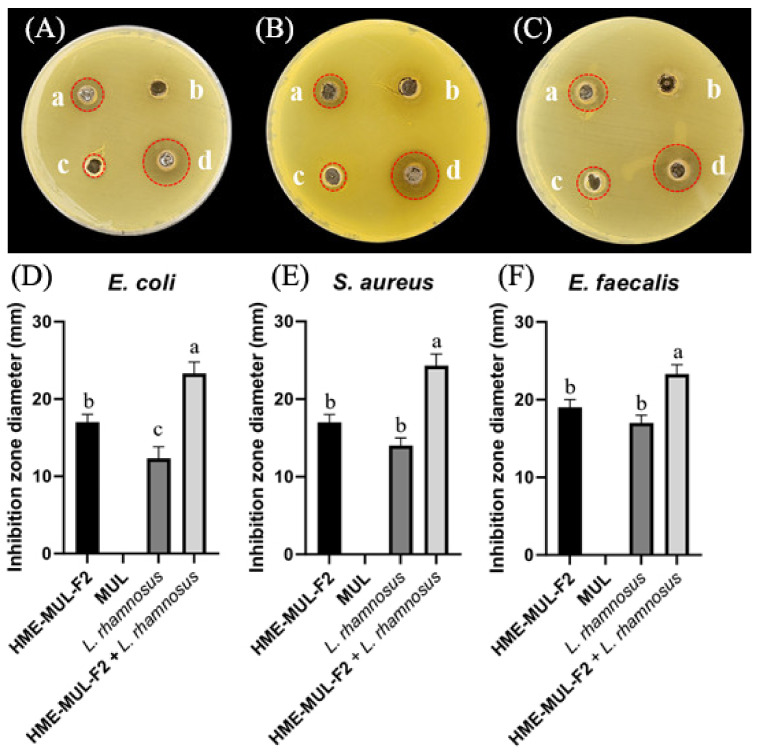
Inhibition zones in the presence of lactic acid bacterial probiotic *L. rhamnosus* against test pathogens after treatment with HME-DDS formulation (HME-MUL-F2). (**A**,**D**): Inhibition against *E. coli*. (**B**,**E**): Inhibition against *S. aureus.* (C) and (F): Inhibition against *E. faecalis*. a: treatment with HME-MUL-F2, b: treatment with MUL, c: treatment with *L. rhamnosus* only, d: treatment with *L. rhamnosus* + HME-MUL-F2. (**A**–**C**) Photos of clear zone in the presence of probiotics, HME-MUL-F2, and MUL. (**D**–**F**) Quantification of inhibition zone diameter in the presence of MUL, HME-MUL-F2, probiotics only, and probiotics + HME-MUL-F2, respectively. a,b value differences in the same condition (*p* < 0.05). Data are presented as mean ± SD (*n* = 3).When *L. rhamnosus* cell suspension alone was used as treatment against pathogenic bacteria *E. coli*, *S. aureus*, and *E. faecalis*, diameters of growth inhibition zones were 13.0 ± 1.0 mm, 18.0 ± 1.0 mm, and 14.0 ± 1.2 mm, respectively. However, when cell suspension of *P. pentosaceus* in combination with HME-MUL-F2 was used as treatment, diameters of growth inhibition zones were 23.0 ± 1.0 mm, 23.3 ± 1.5 mm, and 22.0 ± 1.5 mm, respectively (Figure 11).

MUL, a non-extrusion molded product, did not show antibacterial activity against pathogenic bacteria of *E. coli*, *S. aureus*, or *E. faecalis*. However, when HME-MUL-F2 alone was used for treatment, the activity was 17.0 ± 1.0 mm, 17.0 ± 1.0 mm, and 19.0 ±1.0 mm, respectively. It was confirmed that HME-MUL-F2 had antibacterial activity because a growth-inhibitory region of pathogenic bacteria was formed even after it was used for treatment alone. Even when *L. rhamnosus* cell suspension was used as a treatment alone for the three pathogenic bacteria, diameters of growth inhibition zones were 12.3 ± 1.0 mm, 14.0 ± 1.5 mm, and 17.0 ± 1.0 mm for pathogenic bacteria *E. coli*, *S. aureus*, and *E. faecalis*, respectively, indicating that *L. rhamnosus* culture not treated with HME-MUL-F2 also showed significant antibacterial activities (Figure 10). However, when *L. rhamnosus* cell suspension combined with HME-MUL-F2 was used as treatment, the diameters of growth inhibition zones were 23.3 ± 1.5 mm, 24.0 ± 1.5 mm, and 22.0 ± 1.2 mm for the three pathogenic bacteria *E. coli*, *S. aureus*, and *E. faecalis*, respectively. This confirmed that *L. rhamnosus* cell suspension combined with HME-MUL-F2 showed higher antibacterial activities than the treatment with *L. rhamnosus* or HME-MUL-F2 alone.

## 4. Conclusions

In conclusion, the ability of HME-MUL-F2 to perform sustained release of anthocyanins (ACNs) can regulate the proliferation, metabolism, morphology, and antibacterial activities of probiotics such as *L. rhamnosus* and *P. pentosaceus* and play a role as a regulator of probiotics or prebiotics of natural origin. The results of this study revealed that HME-DDS formulation, HME-MUL-F2, which contained ACNs as an active ingredient, showed antibacterial effects against harmful bacteria distributed in the intestine. It can help maintain the mucosal barrier by preventing the occurrence of harmful intestinal bacterial while maintaining the balance of the entire intestinal microbial flora. Additionally, HME-MUL-F2 is a sustained-release formulation that can continuously release ACNs with a long half-life for each part of the gastrointestinal tract, thus increasing bioavailability.

## Figures and Tables

**Figure 11:**
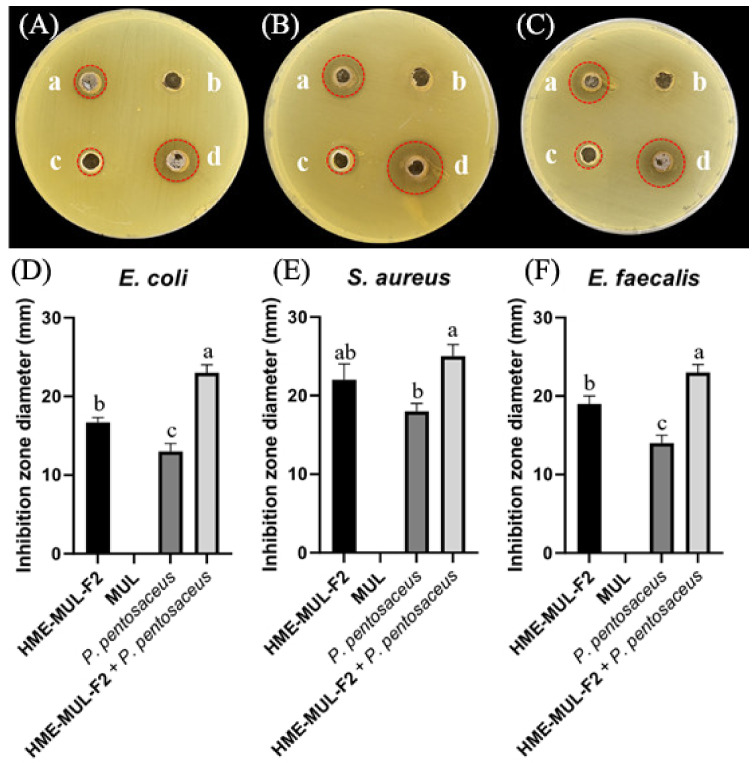
Inhibition zones in the presence of probiotics *P. pentosaceus* against test pathogens after treatment with HME-DDS formulation (HME-MUL-F2). (**A**,**D**): Inhibition against *E. coli.* (**B**,**E**): Inhibition against *S. aureus*. (**C**,**F**): Inhibition against *E. faecalis*. a: treatment with HME-MUL-F2, b: treatment with MUL, c: treatment with *P. pentosaceus* only, d: treatment with *P. pentosaceus* + HME-MUL-F2. (**A**–**C**): Photos of clear zone in the presence of probiotics, HME-MUL-F2, and MUL. (**D**–**F**) Quantification of inhibition zone diameter in the presence of MUL, HME-MUL-F2, probiotics only, and probiotics + HME-MUL-F2, respectively. a,b,c value differences in the same condition (*p* < 0.05). Data are presented as mean ± SD (*n* = 3).Rushdi and Rushdi [72] confirmed that probiotics inhibited the growth of *E. faecalis*, *E. coli*, and *S. aureus* by the paper disc diffusion method. In addition, they have been reported to have antimicrobial activity against pathogenic bacteria, such as *Clostridium perfringens* and *Salmonella typhimurium* [73]. These results indicate that treatment with HME-MUL-F2 further increased the antimicrobial activity of probiotic bacteria. Thus, HME-MUL-F2 might have a prebiotic effect due to its ability to continuously release ACNs.

**Table 1:** Formulation ratio of mulberry and biopolymers (%).

	MUL-HME-F1	MUL-HME-F2	MUL-HME-F3
Mulberry powder	50	50	40
Whey protein isolate	40	-	40
Lecithin	2.5	-	2.5
Ascorbyl palmitate	2.5	5	5
Mannitol	5	35	5
Sodium alginate	2.5	5	5
Poloxamer 188	2.5	5	2.5
Total	100	100	100

**Table 3:** Anthocyanin contents of mulberry extract (ug/g).

		Anthocyanin Content (mg/g DW)	
		C3G	C3R
Raw materials	MUL	43.13 ± 2.63 ^f^	2.99 ± 1.25 ^g^
	MUL-CA	65.07 ± 1.10 ^f^	18.49 ± 0.89 ^g^
	MUL-F1	317.39 ± 18.93 ^c^	136.75 ± 7.97 ^de^
	MUL-F2	289.22 ± 20.75 ^d^	166.12 ± 33.38 ^c^
	MUL-F3	289.13 ± 23.28 ^d^	126.10 ± 7.09 ^d^
Extrusion materials	HME-MUL	117.44 ± 1.44 ^e^	68.91 ± 1.35 ^f^
	HME-MUL-CA	591.62 ± 12.65 ^a^	401.16 ± 13.09 ^a^
	HME-MUL-F1	402.79 ± 6.78 ^b^	188.44 ± 3.03 ^b^
	HME-MUL-F2	325.02 ± 11.12 ^c^	154.73 ± 11.30 ^cd^
	HME-MUL-F3	410.76 ± 13.44 ^b^	164.36 ± 8.46 ^c^

MUL: mulberry; MUL-CA: MUL treated with 0.5 M citric acid; MUL-HME: only solid formulations of the extrudate of MUL; MUL-HME-CA: solid formulations of the extrudate of MUL treated with 0.5 M citric acid. HME-MUL-F1/F2/F3: treatment of mulberry HME-DDS formulation. ^a,b,c,d,e,f,g^ values differences in the same condition (*p* < 0.05). Data are presented as mean ± SD (*n* = 3). The mannitol contained in MUL-HME-F2 exhibits many positive properties in relation to high processing temperature and matrix properties [36]. It forms a hydrophilic layer due to direct hydrogen bonding between proteins. It can also increase the solubility [37]. Therefore, it was confirmed that the reason for the high content of total flavonoids and ACN in MUL-HME-F2 was because particles were stabilized at a high temperature due to mannitol, with solubility increased, leading to a high content. Additionally, it has been reported that poloxamer 188 can protect film loss from heat and reduce damage to components [38]. Total flavonoid, phenol, and ACN contents were melted without breaking. These components were preserved without breaking during extrusion.

**Table 4:** Particle size (nm), PDI, and Zeta potential (mV) of prepared MUL and HME-DDS.

	PSA (nm)	PDI (Index)	ZP (mV)
MUL	329.67 ± 13.37	0.312 ± 0.005	−25.42 ± 4.87
HME-MUL-F1	258.63 ± 21.73	0.325 ± 0.004	−16.76 ± 1.62
HME-MUL-F2	152.03 ± 3.19	0.297 ± 0.013	−31.37 ± 0.24
HME-MUL-F3	218.20 ± 61.48	0.130 ± 0.026	−23.16 ± 1.37

## Data Availability

Data is available within the article or Supplementary Materials.

## Supplementary Materials

The following supporting information can be downloaded at: https://www.mdpi.com/article/10.3390/antiox11112301/s1, Figure S1: Release profile of ANC (C3G and C3R) from PM (physical mixture)-MUL-F1, F2, F3 in gastrointestinal tract simulation.; Figure S2: Lactic acid bacteria and pathogen bacterial growths characteristics of *L. rhamnosus*, *P. pentosaceus*, *S. aureus*, *E. faecalis*, and *E. coli* with correlation of optical density (OD_600_) and colony forming unit.
