# Supplementary material for: Hot-Melt Extrusion Enhances Antioxidant Effects of Mulberry on Probiotics and Pathogenic Microorganisms
Source: Antioxidants (Basel). 2022 Nov 21;11(11):2301. doi: 10.3390/antiox11112301 (PMC9687528; doi:10.3390/antiox11112301)
Supplement: Supplementary file 1 [file antioxidants-11-02301-s001.zip › antioxidants-1993394-supplementary.pdf]

Supplementary Materials

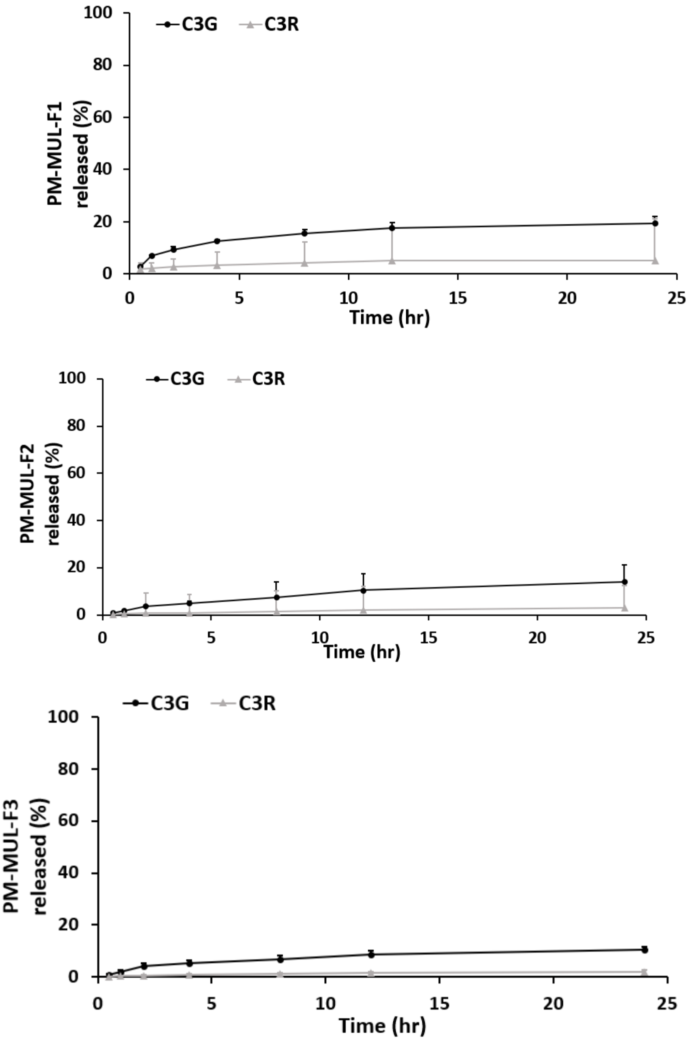

Figure S1. Release profile of ACN (C3G and C3R) from PM (physical mixture)-MUL-F1, F2, F3 in gastrointestinal tract simulation. SGF: simulated gastric fluid, from initial and 2 hr (0, 0.5, 1, and 2 hr), SIF: simulated intestinal fluid from 2 hr to 24 hr (4, 8, 12, and 24 hr).

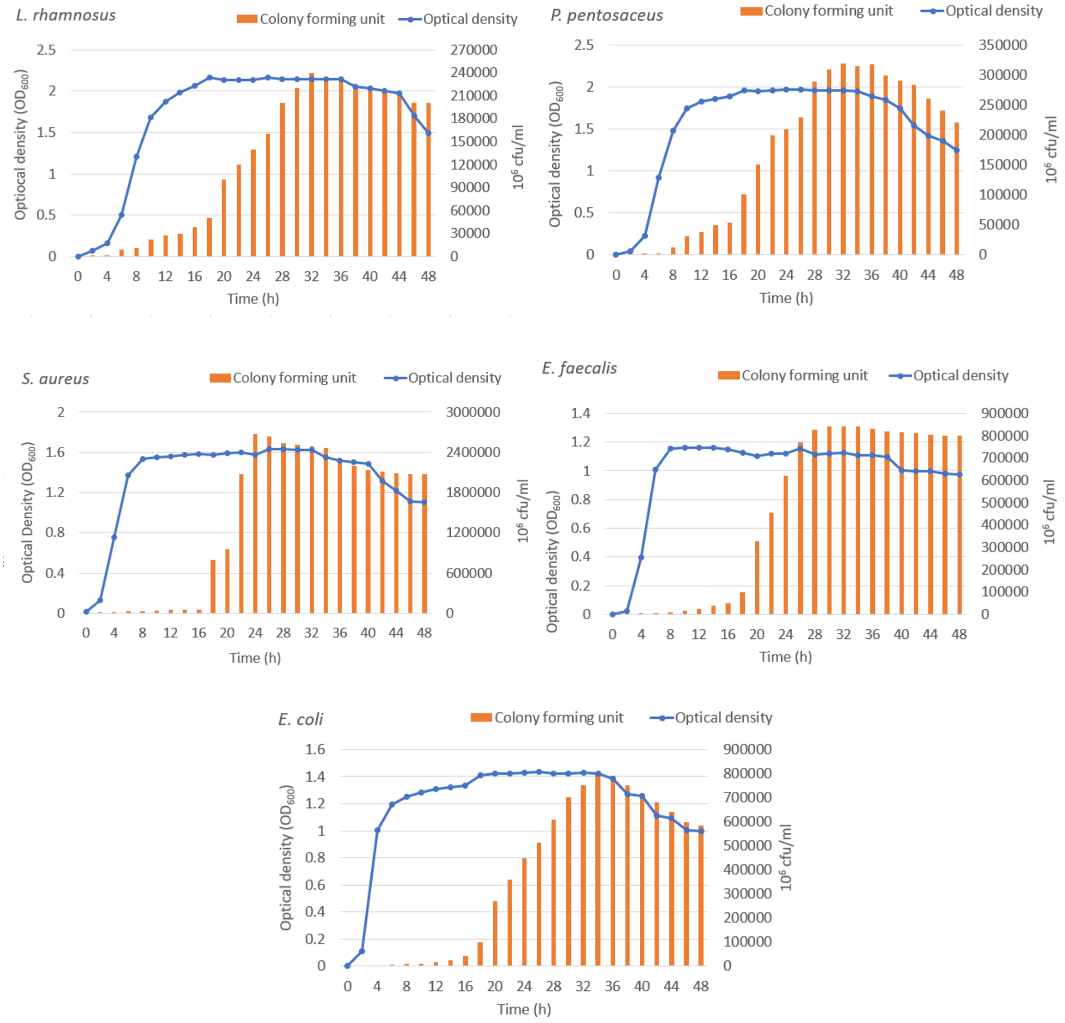

Figure S2. Lactic acid bacteria and pathogen bacterial growths characteristics of *L. rhamnosus*, *P. pentosaceus*, *S. aureus*, *E. faecalis*, and *E. coli* with correlation of optical density (OD_{600}) and colony forming unit.
